# Stable polyp-scene classification via subsampling and residual learning from an imbalanced large dataset

**DOI:** 10.1049/htl.2019.0079

**Published:** 2019-11-26

**Authors:** Hayato Itoh, Holger Roth, Masahiro Oda, Masashi Misawa, Yuichi Mori, Shin-Ei Kudo, Kensaku Mori

**Affiliations:** 1 Graduate School of Informatics, Nagoya University, Furo-cho, Chikusa-ku, Nagoya, 464-8601, Japan; 2 Digestive Disease Center, Showa University Northern Yokohama Hospital, Tsuzuki-ku, Yokohama, 224-8503, Japan; 3 Information Technology Center, Nagoya University, Furo-cho, Chikusa-ku, Nagoya, 464-8601, Japan; 4 Research Center for Medical Bigdata, National Institute of Informatics, Hitotsubashi 2-1-2, Chiyoda-ku, Tokyo, 101-8430, Japan

**Keywords:** feature extraction, image classification, learning (artificial intelligence), cancer, biological organs, computerised tomography, endoscopes, medical image processing, convolutional neural nets, polyp-detection dataset, stable polyp-scene classification method, false positive detection, high-performance CAD system, nonpolyp scenes, colonoscopic video dataset, unstable polyp detection, subsampling, residual learning, imbalanced large dataset, computer-assisted diagnosis system, three-dimensional convolutional neural network, 3D CNN

## Abstract

This Letter presents a stable polyp-scene classification method with low false positive (FP) detection. Precise automated polyp detection during colonoscopies is essential for preventing colon-cancer deaths. There is, therefore, a demand for a computer-assisted diagnosis (CAD) system for colonoscopies to assist colonoscopists. A high-performance CAD system with spatiotemporal feature extraction via a three-dimensional convolutional neural network (3D CNN) with a limited dataset achieved about 80% detection accuracy in actual colonoscopic videos. Consequently, further improvement of a 3D CNN with larger training data is feasible. However, the ratio between polyp and non-polyp scenes is quite imbalanced in a large colonoscopic video dataset. This imbalance leads to unstable polyp detection. To circumvent this, the authors propose an efficient and balanced learning technique for deep residual learning. The authors’ method randomly selects a subset of non-polyp scenes whose number is the same number of still images of polyp scenes at the beginning of each epoch of learning. Furthermore, they introduce post-processing for stable polyp-scene classification. This post-processing reduces the FPs that occur in the practical application of polyp-scene classification. They evaluate several residual networks with a large polyp-detection dataset consisting of 1027 colonoscopic videos. In the scene-level evaluation, their proposed method achieves stable polyp-scene classification with 0.86 sensitivity and 0.97 specificity.

## 1 Introduction

Early detection of polyps in colonoscopies is an essential task for the prevention of colon-cancer deaths. Accurate polyp detection is essential for colonoscopy performance since every 1% increase in the polyp-detection rate can decrease the interval of colorectal cancer incidence by 3% [1]. However, the adenoma-detection rate obtained by colonoscopists varies from 7 to 53%, even though the adenoma-prevalence rate is estimated to be higher than 50% in the screening-age population [2]. The previous meta-analysis also showed that approximately 26% of neoplastic diminutive polyps was missed in a single colonoscopy [3]. Therefore, there is a demand for an image-based computer-assisted diagnosis (CAD) system for colonoscopies to achieve improved colonoscopy performance.

To build a high-performance CAD system, we need an accurate classifier that correctly discriminates polyp-appearing scenes from non-polyp scenes in a sequence of images captured by the colonoscopy. Such stable polyp-scene detection has been a challenge for many years [4–9]. Previous methods are based on two-dimensional (2D) image processing of each still image in a colonoscopic video. MICCAI 2018's endoscopic vision challenge (Endovis18) [6] adopted image-sequence-based evaluation as a detection algorithm by using short colonoscopic videos. In this evaluation, many state-of-the-art methods produced imbalanced results with biased sensitivity and specificity, demonstrating the difficulty of stable polyp detection. Furthermore, in EndoVis18's test dataset, the ratio between polyp and non-polyp scenes was about 2:1. Their evaluation scheme was biased towards sensitivity. Therefore, if we applied the methods submitted to EndoVis18, we may obtain many false positives (FPs). Moreover, state-of-the-art methods [5–9] are variants of object detection methods [10–13] and segmentation methods [14, 15]. Making a large dataset is a notably difficult task for these methods since the training step requires annotation labels for the target object regions in each image.

**Fig. 1:**
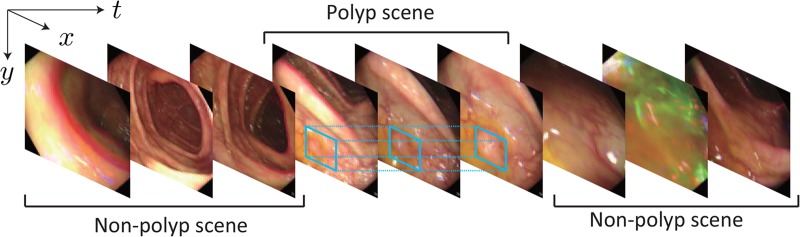
Temporal coherence in polyp-scene classification for polyp detection. As indicated by the blue boxes, the regions of a polyp exist over several frames in a colonoscopic video. This temporal coherence contributes to learning discriminative features for accurate polyp-scene classification

For stable polyp detection, temporal coherence in colonoscopy views is a key structural factor, as shown in Fig. 1. Spatiotemporal feature extraction via a three-dimensional convolutional neural network (3D CNN) [16, 17] utilises temporal coherence in successive colonoscopic images. In previous studies [18, 19], a 3D CNN method achieved about 0.80 sensitivity and specificity in an image-sequence evaluation. These results were attained by a model trained with short videos of only 102 polyps and early stopping.

For further improvement in the detection accuracy of a 3D CNN, we need a larger training dataset. Unlike well-known object-detection [10–13] and segmentation methods [14, 15], assembling a large training dataset is possible for a 3D CNN since it requires only weak annotation, which represents the existence of a polyp in each frame without annotation of the polyp location. However, learning for a 3D CNN with large colonoscopic videos involves an *imbalance*. The ratio between the polyp and non-polyp scenes is quite imbalanced in colonoscopic videos since most scenes do not include a polyp. This property also leads to imbalanced classification results. Furthermore, a 3D CNN is prone to overfitting since it has larger parameters than the usual 2D CNNs.

This paper tackles the imbalance problem of large-scale datasets in deep learning for automated polyp-scene classification in colonoscopic videos. We propose a new residual learning procedure from an imbalanced large dataset. We then introduce post-processing to train models as a way to achieve stable polyp-scene classification.

## 2 Methods

There are roughly two approaches to managing imbalanced datasets in machine learning [20]: using the weighting loss function and manipulating datasets. Where the data size is small, new data is synthesised as data augmentation before the model is trained [21, 22]. However, the synthesising approach is costly when pre-processing a large video dataset. Moreover, generative adversarial networks (GANs)-based data augmentation is premature for real applications for the training of GAN easily diverse and trained GAN outputs images of unstable quality [23, 24]. In unstable output images, some look realistic, and others not. For large-scale machine learning, loss-function weighting in the learning step is a common technique [25, 26]. However, as the epoch continues, the effects of the dataset's imbalance appear. We introduce an online random subsampling technique for large scale deep learning. This reduces both the computational cost and the effect of imbalance.

### 2.1 Class-weight balancing in loss function

For an input query ${\cal X} \in {\opf R}^{T \times H \times W \times 3}$, which is a tensor containing *T*-frames of RGB images, we train the model $f\lpar {\cal X}\rpar $, which returns the scores $\lsqb y_1\comma \; y_2\comma \; \ldots \comma \; y_K\rsqb ^{\rm \top }$ of *K* predefined classes for ${\cal X}$. We introduce a probabilistic representation ${\cal Q} = \lcub Q\lpar l\vert f\lpar {\cal X}\rpar \rpar \rcub _{l = 1}^K $ of the trained model as

(1) $$Q\lpar l\vert f\lpar {\cal X}\rpar \rpar = \displaystyle{{e^{y_l}} \over {\sum\nolimits_{k = 1}^K {e^{y_k}} }}\comma \; \eqno\lpar 1\rpar $$

which is a softmax function with the condition $\sum\nolimits_{l = 1}^K Q\lpar l\vert f\lpar {\cal X}\rpar \rpar = 1$. Using this softmax function, for the *K*-class dataset, we have weighted cross-entropy between the ground truth probabilistic distribution ${\cal P} = \lcub P\lpar l\vert {\cal X}_{n_k}\rpar \rcub _{l = 1}^K $ and the estimated probabilistic distribution ${\cal Q}$ by

(2) $$H_w\lpar {\cal P}\comma \; {\cal Q}\rpar = - \sum\limits_{k = 1}^K \sum\limits_{n_k = 1}^{N_k} w_kP\lpar l\vert {\cal X}_{n_k}\rpar \log \lpar Q\lpar l\vert f\lpar {\cal X}_{n_k}\rpar \rpar \rpar \comma \; \eqno\lpar 2\rpar $$

where *N*, $N_k$ and $w_k = N/KN_k$ are the total number of samples in the train dataset, the number of samples of the train dataset of the *k*th class and a balanced weight [26], respectively. In the learning step, we minimise this weighted cross-entropy. We set $K = 2$ for the two-classes problem: polyp and non-polyp scenes.

### 2.2 Subsampling excepting hard-negative samples

The class-weight balancing approach partly reduces the effect of imbalanced training data with early stopping. However, as the epoch continues, learning with class-weight balancing leads to overfitting of the dominant category, which contains most of the population in the training data.

For large-epoch training, we design a new subsampling approach. In the two-category classification of positive and negative categories as in polyp-scene classification, we assume that negative samples are the dominant category. In this large set of negative samples, we obtain specific samples, which include difficult patterns for correct classification, as hard-negative samples. We use hard-negative samples as the default set and randomly select a subset of the residual negative samples at each epoch in deep learning so that the ratio of positive and total negative samples is 1:1. We assume that these hard-negative samples distribute around the decision boundary between the two categories. Therefore, this procedure selects an important pattern for training and utilises all patterns in the negative samples after long learning with a balanced ratio of the two-category population.

### 2.3 Architecture for polyp-scene classification

**Fig. 2:**
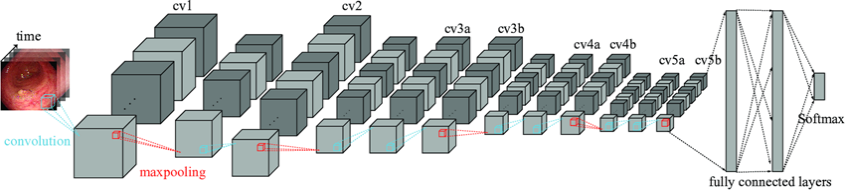
Architecture 3D CNN (C3DNet [16]) for polyp-scene classification. We added batch normalisation to the convolutional layers and added a dropout of 0.5 to the fully connected layers

We adopt spatiotemporal feature extraction with deep learning for our polyp-scene classification. In our previous work, we used a 3D CNN, C3DNet, for the spatiotemporal feature extraction [18]. C3DNet is a natural extension of 2D CNNs for 3D data classification [16], as shown in Fig. 2. However, C3DNet is prone to early convergence and overfitting. In our previous work [18], we constructed a C3DNet model with early stopping and class-weight balancing to avoid overfitting of non-polyp scenes. Our C3DNet model achieved 0.80 sensitivity and specificity for the test data extracted from the videos, where only typical protruding polyps appear.

Residual learning is a state-of-the-art method to avoid overfitting and extract fine features for classification. Therefore, we adopt a 3D version of the residual network (3D ResNet) to avoid overfitting [17]. In addition to introducing residual units, we introduce a wide residual architecture (Wide ResNet) [27] by extending from a 2D convolution to a 3D convolution. The Wide ResNet doubles the number of kernels of the usual ResNet at the downsampling step. We set the weight decay to be 0.0001 for all residual units in our architectures and selected different sizes, numbers, and strides of kernels in the convolution to optimise the architectures for polyp-scene classification. However, the number, size, and stride width of the convolutional kernel and pooling are basically inherited from the C3DNet of our previous works [18, 19]. Table 1 summarises the architecture of our 3D ResNets. Models II and IV are wider versions of models I and III, respectively. In these architectures, the number of parameters of the 3D CNN and 3D ResNet is about 28 million and 14 million, respectively. To the best of our knowledge, this is the first time, 3D ResNet has been applied to polyp-scene classification.

**Table 1:** Architect of our 3D ResNet for polyp detection

Layer name	Architecture			
	I	II	III	IV
conv1	$3 \times 3 \times 3\comma \; \; \; 32$, stride $1 \times 1 \times 1$		$3 \times 3 \times 3\comma \; \; \; 64$, stride $1 \times 1 \times 1$	
	max pool, stride $1 \times 2 \times 2$	max pool, stride $2 \times 2 \times 2$	max pool, stride $2 \times 2 \times 2$	max pool, stride $2 \times 2 \times 2$
conv2_*	$\left[{\matrix{ {3 \times 3 \times 3\comma \; } & {32} \cr {3 \times 3 \times 3\comma \; } & {32} \cr } } \right]$	$\left[{\matrix{ {3 \times 3 \times 3\comma \; } & {64} \cr {3 \times 3 \times 3\comma \; } & {64} \cr } } \right]$	$\left[{\matrix{ {3 \times 3 \times 3\comma \; } & {64} \cr {3 \times 3 \times 3\comma \; } & {64} \cr } } \right]$	$\left[{\matrix{ {3 \times 3 \times 3\comma \; } & {128} \cr {3 \times 3 \times 3\comma \; } & {128} \cr } } \right]$
conv3_*	$\left[{\matrix{ {3 \times 3 \times 3\comma \; } & {64} \cr {3 \times 3 \times 3\comma \; } & {64} \cr } } \right]$	$\left[{\matrix{ {3 \times 3 \times 3\comma \; } & {128} \cr {3 \times 3 \times 3\comma \; } & {128} \cr } } \right]$	$\left[{\matrix{ {3 \times 3 \times 3\comma \; } & {128} \cr {3 \times 3 \times 3\comma \; } & {128} \cr } } \right]$	$\left[{\matrix{ {3 \times 3 \times 3\comma \; } & {256} \cr {3 \times 3 \times 3\comma \; } & {256} \cr } } \right]$
conv4_*	$\left[{\matrix{ {3 \times 3 \times 3\comma \; } & {128} \cr {3 \times 3 \times 3\comma \; } & {128} \cr } } \right]$	$\left[{\matrix{ {3 \times 3 \times 3\comma \; } & {256} \cr {3 \times 3 \times 3\comma \; } & {256} \cr } } \right]$	$\left[{\matrix{ {3 \times 3 \times 3\comma \; } & {256} \cr {3 \times 3 \times 3\comma \; } & {256} \cr } } \right]$	$\left[{\matrix{ {3 \times 3 \times 3\comma \; } & {512} \cr {3 \times 3 \times 3\comma \; } & {512} \cr } } \right]$
conv5_*	$\left[{\matrix{ {3 \times 3 \times 3\comma \; } & {256} \cr {3 \times 3 \times 3\comma \; } & {256} \cr } } \right]$	$\left[{\matrix{ {3 \times 3 \times 3\comma \; } & {512} \cr {3 \times 3 \times 3\comma \; } & {512} \cr } } \right]$	$\left[{\matrix{ {3 \times 3 \times 3\comma \; } & {512} \cr {3 \times 3 \times 3\comma \; } & {512} \cr } } \right]$	—
conv6_*	$\left[{\matrix{ {3 \times 3 \times 3\comma \; } & {512} \cr {3 \times 3 \times 3\comma \; } & {512} \cr } } \right]$	—	—	—
global average pooling and softmax.				

Each square bracket represents a residual unit, which is comprised of conv*i*_1, batch normalisation, ReLu, conv*i*_2, batch normalisation, addition, and ReLu, in this order [17].

### 2.4 Post-processing

In real colonoscopic scenes, inappropriate still images for diagnosis can be captured, such as images that are out of focus and/or blurred due to the colonoscope moving too fast. Moving the colonoscope too quickly also results in some still images in which the polyps are out of sight. Other examples include occlusion by the colon wall and bubbles that conceal polyps in the polyp scenes. When the colonoscope moves too quickly, these inappropriate still images can appear in the successive images in polyp scenes. These images are a kind of noise and lead to misclassification in 3D CNN. Therefore, we use post-processing for the trained models. We set criteria $\tau $. If a trained model outputs a value larger than $\tau $ over *N*-successive samples in an input video, the trained model outputs a polyp-scene label. This post-processing reduces FPs and preserves true positives (TPs).

## 3 Experiments and results

### 3.1 Dataset

We constructed a new dataset to validate our proposed learning method. We collected a total of 1027 full-HD-resolution 30-frames-per-second (fps) videos of 951 patients captured by the CF-HQ290ZI (Olympus, Tokyo, Japan) during a daily colonoscopy at Showa University Northern Yokohama Hospital with IRB (institutional review board) approval. We annotated all of the videos’ frames via two-step procedures with the annotation tool ELAN [28]. In the first step, two trained support staff performed annotations for all of the frames of each video. In the second step, two expert colonoscopists checked the annotated videos provided by the support staff. The annotation labels were the polyp existence, gross anatomy, polyp size, and observation conditions for all frames in the videos. The gross anatomy consists of the types of polyps (Is, Ip, Isp and IIa) and the observation conditions are white light, narrow-band imaging, and staining modes. These gross anatomical annotations suggest that both protruding and flat type polyps exist in our dataset. Note that about 60% of polyps in our dataset are the flat type. The annotated videos are divided into two groups: polyp and non-polyp videos. In this evaluation, we used the polyp existence and observation condition labels. Note that we used only scenes captured under the white light condition.

We divided these videos into the training, validation, and test datasets without duplication of the patients among them. The two expert colonoscopists selected typical polyp examples for detection by the CAD system for the validation and test datasets. The training, validation, and test datasets include both protruding and flat polyps. We used the validation data to check for overfitting in training and to select the trained model for evaluation. We used the test data to evaluate the trained model, which was selected by checking the validation-data results. Note that there is no duplication in the divided data. Therefore, we evaluated our trained model with unseen images. Furthermore, we selected hard-negative videos. We are already aware of difficult scenes in colonoscopic videos for polyp-scene classification via our previous works [18, 19]. In these difficult scenes, we observed specific colonoscope actions, such as moving too close to and stopping near the colon walls. When colonoscopists capture the shapes of polyp-like structures, they bring the colonoscope close to the colon walls and stop it. These actions in non-polyp scenes lead to FPs because the 3D CNN recognises the actions in videos. In addition to these actions, the appearance of the specula and bubbles sometimes leads to FPs. Therefore, we selected non-polyp videos that include specula and bubbles.

We then extracted 16-frame chunks from each dataset. This extraction allowed for an overlap of eight frames between two successive chunks. The chunk size was $16 \times 112 \times 112 \times 3$ (frames × height × width × channels), which is the common size in 3D convolution-based spatiotemporal feature extraction [16, 17, 29]. Our previous works also used this size [18, 19]. Table 2 summarises the details of our dataset.

**Table 2:** Summary of our polyp detection dataset

	Polyp videos				Non-polyp videos			
	Train	Validation	Test	Total	Train	Validation	Test	Total
#patient	745	24	24	793	154	12	13	158
#videos	797	31	40	868	165	15	11	159
#polyp	1945	31	41	2017	0	0	0	0
#chunks	90,096	2274	2184	94,554	284,510	21,436	20,291	326,237

# patients represents the number of patients. # videos represents the number of videos captured from the patients. # polyps and # chunks counts the number of polyps in the videos and chunks extracted from the videos, respectively. The length of each video is from 10 to 25 min. In the set of non-polyp videos, 32 videos of 21 hard-negative samples, which were difficult non-polyp scenes for classification, provided 46,086 chunks.

### 3.2 Training of models

We trained a 3D CNN and 3D ResNet for our polyp-scene classification. We used an NVIDIA Tesla V100 16 GB GPU and a Keras with a TensorFlow backend. For the C3DNet, we trained two models with balanced weighting and our random subsampling, respectively. For these two trainings, we set an Adam optimisation algorithm with a default learning rate of lr = 0.00001 for 30 epochs. For the 3D ResNet, we used our random subsampling set at lr = 0.000001 for 70 epochs. We selected the best-trained models for each architecture by checking the classification accuracy with the validation data. These base learning rates were decided via preliminary experiments by checking the learning curves and selecting base learning rates that moderately lose values of loss functions for both the training and validation data.

### 3.3 Chunk-level evaluation

**Fig. 3:**
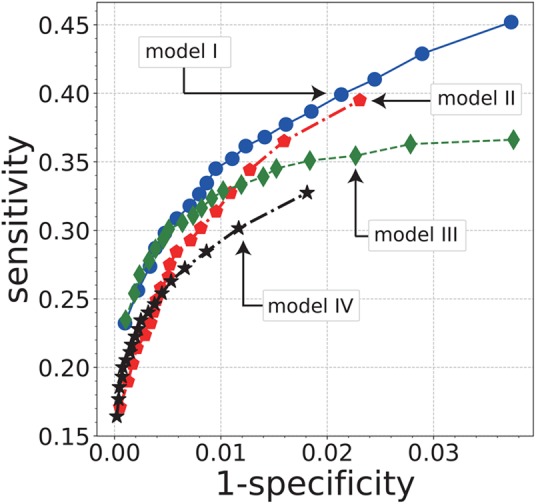
ROC curves of polyp-scene classification in four models. For this plotting, we set $\tau = 0.05\comma \; 0.10\comma \; \ldots \comma \; 0.95$ as the decision criteria for the softmax output against the polyp scene. The vertical and horizontal axes represent sensitivity and 1-specificity, respectively

Using the trained models of each architecture, we computed the sensitivity and specificity against the test data. Sensitivity and specificity are the ratios of TPs in the polyp and non-polyp scenes, respectively, of the test data. Fig. 3 illustrates the evaluation results of the ResNets in a chunk level. We omitted the results of two C3DNets, trained with class-weight balancing and our subsampling, respectively, since both models had almost zero sensitivity. Note that the C3DNet trained with our random samples gives higher sensitivity than the C3DNet trained with class-weight balancing for evaluation against the training data, even though both models are overfitted to the training data.

### 3.4 Scene-level evaluation

**Fig. 4:**
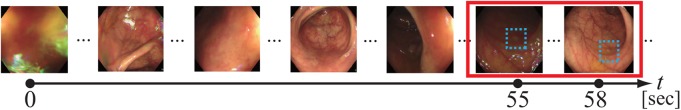
Example of short 60-second video clips for scene-level evaluation. The red square designates a polyp scene. In the polyp scene, several frames are blurred because the colonoscope moved quickly. The blue-dashed lines indicate the polyp locations of each frame. Non-polyp scenes include bubble scenes and blurry scenes. The non-polyp scenes include scenes that are too close to the colon wall

We performed a scene-level evaluation of the trained ResNets using 21 short video clips of 50–60 seconds. We created these 21 video clips from 40 polyp videos of 41 polyps in the test data. Each clip includes polyp scenes of one polyp and the length of one polyp scene is from 2 to 10 seconds. These clips were cropped from videos of actual endoscopies. Therefore, these polyp scenes include inappropriate still images due to quick colonoscope movement with bubbles and specula. This allows for practical evaluation as such inappropriate images inhibit polyp detection. Fig. 4 shows an example of these 21 short video clips.

We fed these 21 clips to the four trained models with post-processing, where we extracted 16-frame chunks with 4-frame overlap between successive chunks. For post-processing, we set decision criteria $\tau = 0.5$ for all four trained models. We chose these criteria to compare the four models in the same setting. We set *N* in post-processing to be 2 and 3 for models I and III, and II and IV, respectively. This setting of *N* was decided from the validation data classification.

**Fig. 5:**
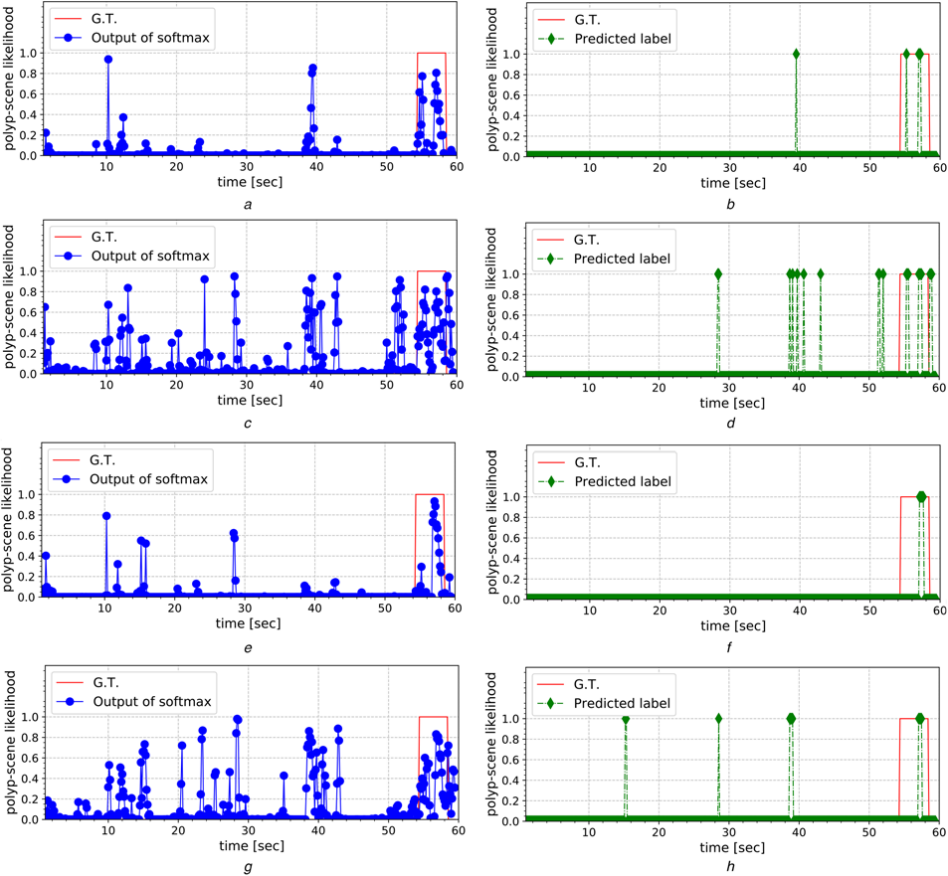
Examples of qualitative evaluation. (a), (c), (e), and (g), and (b), (d), (f), and (h) summarise the softmax outputs and prediction labels for the short video clips shown in Fig. 4, respectively. From top to bottom, the first to fourth rows summarise the results given by models I, II, III, and IV, respectively. Note that the inputs for the models are sets of 16 frames (chunks) with a 4-frame overlap in successive chunks *a* Model I *b* Model I *c* Model II *d* Model II *e* Model III *f* Model III *g* Model IV *h* Model IV

In this evaluation, we conclude that correct detection is performed if the trained model detects at least one chunk in a polyp scene of a short video clip. We set the detection accuracy to be the ratio of correctly detected polyps in the 21 short video clips. Fig. 5 shows an example of this evaluation. In Figs. 5*b*, *d*, *f* and *h*, a polyp is correctly detected since the trained models classified several chunks for an approximately four-second polyp scene, as marked by the red lines. In addition to detection accuracy, we computed the average length of FP scenes among these 21 clips. FP scenes are non-polyp scenes that are incorrectly classified as polyp scenes. Table 3 summarises the detection accuracy and average length of the FP scenes.

**Table 3:** Results of the scene-level evaluation

Model	Detection accuracy	Average length of FP scenes, s
I	0.86	1.5
II	0.95	6.0
III	0.71	0.8
IV	1.00	4.5

## 4 Discussion

In the results of the chunk-level evaluation shown in Fig. 3, our ResNets with proposed random sampling reduced the overfitting of the non-polyp scenes, even though the C3DNet resulted in overfitting of zero sensitivity. Furthermore, random sample preserving of hard-negative samples achieved a high specificity of about 0.99. In the results in Fig. 3, the sensitivities of the trained models appear insufficient. However, the polyp scenes in the test data include many inappropriate noise images for the polyp-scene classification since the test data is sampled from actual colonoscopic videos. Furthermore, the inputs of the models partly include non-polyp scenes at the beginning and the end of the polyp scenes because the inputs in the evaluation are sets of 16 frames with an 8-frame overlap in successive chunks. Therefore, the test data is a very difficult set of practical-diagnosis scenes.

In the results in Fig. 5, we observe FPs even though we obtained high specificity in Fig. 3. In the chunk-level evaluation, we extracted chunks of polyp and non-polyp scenes from the polyp and non-polyp videos, respectively. However, in the scene-level evaluation, we extracted successive chunks of polyp and non-polyp scenes from the polyp videos. Therefore, non-polyp scene chunks include colon wall surface textures similar to the polyp scene textures. These similar textures sometimes lead FPs.

The results of the scene-level evaluation demonstrate the validity of our post-processing. In the examples shown in Fig. 5, the post-processing reduced many FPs and retained TPs of polyp scenes. The bubble and blurry scenes shown in Fig. 4 were rejected through our post-processing. There are FPs after post-processing in Figs. 5*d* and *h*. Models II and IV have higher representation ability of pattern than models I and III because they have the wide residual architecture of models II and IV. Therefore, models II and IV provide more FP scenes that have textures similar to the polyp scenes.

As summarised in Table 3, model II achieved the highest detection accuracy in the scene-level evaluation. However, the average length of the FP scenes was 6 seconds in model II, which is about 10% of the short video clips. This implies that model II is unstable. Model I achieved the second highest detection accuracy with the second shortest average length of FP scenes. This average length accounts for only about 3% of the short video clips. In other words, model I achieved 0.86 sensitivity and 0.97 specificity in the scene-level evaluation, indicating that model I achieves stable polyp detection. We showed the detection example in Fig. 5 to three expert colonoscopists, who reported that model I appears to be the most practical model for real colonoscopy use.

## 5 Conclusions

Towards practical polyp-scene classification in colonoscopic videos, we tackled the imbalance problem of large-scale datasets in deep learning by establishing a new residual learning procedure from an imbalanced large dataset. We then introduced post-processing to the trained models as a way to achieve stable polyp-scene classification. We validated our proposed residual learning procedure and post-processing through experiments with a large dataset of 1027 colonoscopic videos. Our proposed method established stable polyp-scene classification in the scene level of an actual colonoscopic video with a moderate sensitivity of 0.86 and high specificity of 0.97. We conclude that this stable polyp-scene classification is helpful in real colonoscopies as a practical CAD system.

## 6 Funding and declaration of interests

Parts of this research were supported by AMED (19hs0110006h0003), MEXT KAKENHI (26108006, 17H00867, 17K20099), and the JSPS Bilateral Joint Research Project. Conflict of interest: None declared.
